# 

**Published:** 1909-09

**Authors:** 


*The following donations have been received since the publication of the List in June.*

*August* 31*st*, 1909.

J. B. Hurry, M.D. (1) .. .. .. .. .. .. .. 1 volume.
The Middlesex Hospital (2) .. .. .. .. .. .. 1 ,,
James Willing, Jun., Ltd. (3) .. .. .. .. .. .. 1 ,,

### SEVENTY-THIRD LIST OF BOOKS.

The titles of books mentioned in previous lists are not repeated.

The figures in brackets refer to the figures after the names of the donors, and show by whom the volumes were presented. The books to which no such figures are attached have either been bought from the Library Fund, or received through the *Journal*.

**Allbutt and H. D. Rolleston, Sir C.** [Eds.]. *A System of Medicine.* Vol. V 1909

**Archibald, A. Balfour and R. G.** *Review of Recent Advances in Tropical Medicine* .. .. .. .. .. .. .. 1908
**Balfour and R. G. Archibald, A.** *Review of Recent Advances in Tropical Medicine* .. .. .. .. .. .. .. 1908
**Burghard, F. F.** [Ed.]. *A System of Operative Surgery.* Vol. IV.. 1909
**Burton-Fanning, F. W.** *Open-Air Treatment of Pulmonary Tuberculosis* .. .. .. .. .. .. 2nd Ed. 1909
**Collier, W.** .. .. *School Athletics and Boys' Races* .. .. .. 1909
**Cope, V. Z.** .. .. *Minor Gynæcology* .. .. .. .. .. .. .. 1909
**Dawson, Grace** .. *How to Rest and be Rested* .. .. .. .. 1909
**Emery, W. D'E.** .. *Immunity and Specific Therapy* .. .. .. 1909
**Folsom, C. F.** .. *Studies in Criminal Responsibility* .. .. 1909
**Hart, A. H.** .. .. *How to Cut the Drug Bill* .. .. .. .. .. 1909
**Hurry, J. B.**.. .. *A History of the Reading Pathological Society* .. .. .. .. .. .. .. .. (1) 1909
**Jennings, O.** .. *The Re-education of Self-Control in the Morphia Habit* .. .. .. .. .. .. .. .. .. .. 1909
**Jones, R. L.** .. *Arthritis Deformans* .. .. .. .. .. .. .. 1909
**Lockwood, C. B.** .. *Aseptic Surgery* .. .. .. .. .. .. 3rd Ed. 1909
**Mackenzie, J.** .. *Symptoms and Their Interpretation* .. .. 1909
**Neumann, H.** .. *Otitic Cerebellar Abscess.* (Tr. by R. Lake).. 1909
**Pye, [W.]** .. .. *Surgical Handicraft.* (Ed. by W. H. Clayton-Greene) .. .. .. .. .. .. .. 5th Ed. 1909
**Rolleston, Sir C. Allbutt and H. D.** [Eds.]. *A System of Medicine.* Vol. V 1909
**Savill, T. D.**.. .. *Lectures on Hysteria* .. .. .. .. .. .. 1909
**Symes, J. O.**.. .. *Parents, Teachers and Schools* .. .. .. 1909

### TRANSACTIONS, REPORTS, JOURNALS, &c.

American Association of Obstetricians and Gynecologists, Transactions of the .. .. .. .. .. .. .. .. .. Vol. XX 1908

American Medicine .. .. .. .. .. .. .. .. N.S., Vol. III 1908

American Pediatric Society, Transactions of the .. Vol. XX 1909

Annales de Dermatologie et de Syphiligraphie .. .. .. Tom. IX 1908

Archives of the Middlesex Hospital .. .. .. .. .. (2) Vol. XV 1909

Bristol Port Sanitary District, Annual Report of Medical Officer for 1908

British Medical Journal, The .. .. .. .. .. .. .. Vol. I for 1909

Bulletin de l'Académie de Médecine .. .. .. .. Tom. LIX, LX 1908

Bulletin de l'Académie royale de Médecine .. .. .. Tom. XXII 1908

Bulletin de la Société française de Dermatologie.. .. .. .. .. 1908

Census of the British Empire .. .. .. .. .. .. .. .. 1906

Clinical Journal, The .. .. .. .. .. .. .. Vol. XXXIII. 1906

Hospitals—

City Hospital, New York, Medical and Surgical Report Vol. I 1909

St. Thomas's Hospital Reports .. .. .. .. .. Vol. XXXVI 1909

Journal of Hygiene, The .. .. .. .. .. .. .. Vol. VIII 1908

Journal of the American Medical Association, The .. .. Vol. LI 1908

Lancet, The .. .. .. .. .. .. .. .. .. .. Vol. I for 1909

Medical Chronicle, The .. .. .. .. .. .. N.S., Vol. XVI 1908–09
Nordiskt Medicinskt Arkiv .. .. .. .. .. .. .. .. .. .. .. .. 1907
Northumberland and Durham Medical Journal .. .. .. .. .. 1907–08
Post-Graduate, The .. .. .. .. .. .. .. .. Vol. XXIII 1908
Progrès médical, Le .. .. .. .. .. .. .. .. .. .. .. .. .. 1908
Public Health Reports issued by the Surgeon-General of the Public Health and Marine Hospital Service Vol. XXIII, Pt. 1 1908
Royal Academy of Medicine in Ireland, Transactions of the Vol. XXVII 1909
Willing's Press Guide .. .. .. .. .. .. .. .. .. .. .. .. (3) 1909
Wellcome Research Laboratories, Third Report of the .. .. .. .. 1908

---

### Local Medical Notes.

**University of Bristol.**—FACULTY OF MEDICINE :—

EXAMINATION SUCCESSES.

F.R.C.S.ENG.—Edmund A. Dorrell, D.P.H., Walter R. Battye, Capt. I.M.S.

UNIVERSITY OF LONDON.—*First Examination for Medical Degrees:* H. J. Orr-Ewing, John R. Harris, W. E. Taylor. *Distinguished in Physics:* C. C. Harrison, E. S. Sowerby. *Second Examination for Medical Degrees—Part I:* G. M. Campbell, Alice H. Christie, E. W. Wade.

M.B., B.S.LOND.—*Intermediate Examination:* R. M. Wright, R. F. Bolt.

M.D.LOND.—(*Diseases of Women*) *University Medal:* Henry Devine.

CONJOINT BOARD OF ENGLAND.—*Chemistry and Physics:* C. L. Emmerson, J. W. Gilbert, C. J. D. May, C. N. Vaisey, P. Wynn-Wernick. *Biology:* C. N. Vaisey. *Pharmacy:* R. C. Clarke, C. Kingston, W. A. Reynolds. *Anatomy and Physiology:* G. F. Fawn, W. E. Neale. *Surgery:* F. C. Morgan, B. W. A. Stone.* *Midwifery:* B. C. Eskell, R. S. Statham.

M.R.C.S., L.R.C.P.LOND.—John M. Hammond, M.B., B.S. Lond.

L.D.S.ENG.—*Chemistry and Physics:* R. H. Basker, P. W. Flook, E. J. Handford.

D.P.H.LOND.—Thomas E. Holmes, M.D., B.C.Cantab.

APPOINTMENTS.

Smith, G. Munro, M.R.C.S.—Consulting Surgeon at Bristol Royal Infirmary.

* Qualified.

Mole, H. F., F.R.C.S.—Surgeon at Bristol Royal Infirmary.

Walters, C. F., F.R.C.S.—Assistant-Surgeon at Bristol Royal Infirmary.

Clarke, Professor J. Michell, M.D.—Pro-Vice-Chancellor of the University of Bristol.

Hill, Walter J., L.R.C.P.Lond., M.R.C.S.—Medical Officer of Health of Clevedon, Som.

**R.A.M.C. 3rd South Midland Field Ambulance. Presentation of a Drum-Major's Staff.**—This interesting ceremony took place on July 9th at the Drill Hall, when Brigadier-General H. A. Raitt, C.B., addressing the corps, said he had much pleasure in presenting them with a drum-major's staff on behalf of the medical profession of Bristol. It gave him great pleasure for two reasons—first, because it showed the great interest which the medical profession had taken in the formation of that corps, and also because that corps deserved some special recognition, because it was the first field ambulance in England to be complete up to its full establishment. That ought to be a matter of pride not only to the corps and the medical profession, but also to the whole of Bristol.

Dr. Shingleton Smith, on behalf of the medical profession, thanked Brigadier-General Raitt for coming there and performing this duty, and for the remarks he had made. After giving a few particulars in connection with the formation of the corps, he spoke of its duties, which was to bring patients alive to the hospital and in the most expeditious way possible. They were all gratified at the way this corps had qualified itself for its work. Lieut.-Colonel Prichard and his brother officers had been so successful that the corps, although only a year old, was in a state of great efficiency. They had had good training in field service work and stretcher drill, and also some experience of camp life. The staff presented that evening had been given by the medical profession to mark their appreciation of the hard work done by the medical officers associated with the corps. But not only was credit due to the officers, but also to the men, who, after a day's work, were willing to sacrifice their evenings and come to drill. This they had done cheerfully for the love of the work and from a sense of duty to their country.

Mr. Richardson Cross said that as citizens they all felt gratified that Mr. Haldane's great scheme for the improvement of the army in this country seemed to be going on towards a thoroughly satisfactory conclusion, and as medical men they felt glad that the work done in the direction of the medical corps should also be going on well. It was because Colonel Prichard and his officers and men had worked so successfully that they thought some little personal recognition should be paid them by the medical profession, to show how interested they were in this movement. It was also a pleasure to feel that the ambulance and hospital arrangements all over the country were going forward in a satisfactory manner.

Lieut.-Colonel PRICHARD thanked Brigadier-General Raitt for coming there that day and making that presentation, and those who were there for encouraging them in the work they were doing by their presence. He also sincerely thanked the medical profession for the interest they have taken in them, and for their acceptable gift, and in conclusion referred to the creditable manner in which the men had given up their evenings to learn their drill.

**Typhoid Fever Convalescents.**—Recently acquired knowledge as to the efficiency of continuously infective persons (generally known as chronic typhoid " carriers "), in the maintenance and distribution of typhoid fever infection, suggests that physicians dealing with cases of typhoid fever, especially in public Institutions, might do well to arrange to keep a *register of convalescents* and their addresses with the view of securing their periodic attendance after convalescence for a period of six months in order to determine bacteriologically their actual condition as to infectivity.

The result of the inquiry by the Indian Government into the persistence of typhoid fever in the British Army in India has been to confirm very definitely the importance of the " carrier " case, and the investigators confidently declare that the problem of typhoid fever prevention amongst the British troops in India consists in the detection and control of the " carrier," especially in regard to participation in food preparation.

The ultimate aim must of course be to discover a method of cure, but in the meantime the circulation of simple rules amongst convalescent patients may lead to some diminution of danger.

The following provisional rules are suggested with a view to diminish the risk of infection :—

(1) The hands and nails should be thoroughly washed, first in disinfectant solution, then in soap and water, and well rinsed before touching any foodstuffs, especially milk.

(2) After the bowels are opened or the bladder is emptied the hands and nails should be at once disinfected and washed.

(3) Strong disinfecting solution should be poured over the stools, etc., before the plug of the water-closet is used or the lid of the dry closet is shut down.

(4) When the motions are loose the risk of infection is increased and additional care should be taken.

(5) The convalescent should periodically visit the medical attendant, so that the blood, etc., may be tested, and measures may be taken to control the action of the germs. As there is generally no need to deal with the drains, or to disinfect rooms or houses for these special cases, no upset of the domestic arrangements is necessary.

(6) In the household where a patient is convalescing from typhoid fever the additional precautions of boiling all the milk or water used for drinking or in the preparation of food should be undertaken, and uncooked foods should be avoided.

**Antitoxin Treatment in Diphtheria** (prepared for the guidance of medical practitioners chiefly from a paper by Dr. Claude B. Ker, M.D., F.R.C.P.—*The Practitioner*, January, 1909).

*Treatment.*—The keynote of success in the treatment of diphtheria is the early administration of antitoxin. If every case could be injected with antitoxin on the first day of illness, and kept at rest for three weeks, very favourable results would ensue. Spread of the membrane is rare after the injection of antitoxin; but the chances of recovery grow less with each day of postponement. The importance of early dosage cannot be overestimated. In any but the mildest of throats, the result of culture should not be awaited, but injection of antitoxin should be made at once if the clinical symptoms warrant the clear suspicion of diphtheria. Medical practitioners should notify at once, specifying urgency, by telephone if necessary, when the diagnosis is clinically certain and removal urgent. Removal will then be made, and the culture, which should be taken in every case, can be dealt with later.

*Prophylacic Doses.*—Even a small does of 2,000 units may be of much service, if it is necessary to await the result of a culture before removal can be definitely arranged for, and this dose cannot harm the patient.

*Carriers.*—If diphtheria breaks out in a school or institution all throats and noses should be at once tested for the bacillus; similarly if cases succeed one another in a family in spite of removal of patients to hospital and disinfection, the throats and noses of other members of the family should be at once tested for the bacillus, which will be found to be carried by people and not by drains. If prophylactic doses are necessary amongst contacts, 500 units will act as an efficient protection for three weeks, but it must be borne in mind that, though this is a protection to the individual, it does not prevent him becoming a "carrier" to others.

*Dose.*—By age—Infants—not more than 4,000 units at one dose. Children over one year—6,000 units may be given in marked cases. (As children suffer from diphtheria more severely than adults, the necessary doses are comparatively greater.) Adults—8,000 units as a rough maximum for one dose. Minimum dose—2,000 units, for example in a case which is more bacteriological than clinical diphtheria.

DOSE ACCORDING TO SITUATION AND EXTENT OF LESION.

TABULAR GUIDE TO INITIAL DOSE OF ANTITOXIN REQUIRED.

| | Tonsillar lesions only | Pillars of Fauces also involved | Uvula also involved | Posterior Pharyngeal wall also involved | Laryngeal Symptoms also | Nose involved also | |
|---|---|---|---|---|---|---|---|
| 1st day | 1000 | 2000 | 3000 | 4000 | 6000 | 8000 | UNITS |
| 2nd day | 3000 | 4000 | 5000 | 6000 | 6000 | 8000 | |
| 3rd day | 4000 | 5000 | 6000 | 7000 | 6000 | 8000 | |
| 4th day | 5000 | 6000 | 7000 | 8000 | 6000 | 8000 | |

In purely laryngeal cases at least 6,000 units should be given, whether dyspnœa is present or not. Nasal cases with actual membrane and toxic symptoms require full doses. Nasal cases with merely a chronic dirty discharge and no toxic symptoms may require little. Any cases presenting evidence of much toxæmia require full doses.

*Indications for repetition of Dose.*—If case is treated on first day, second dose may not be necessary. If no improvement in symptoms or amount of membrane, repeat in twelve hours. If symptoms are less urgent and some improvement has resulted, interval may be extended to twenty-four hours, but if patient is extremely ill, dose should be repeated in eight hours.

*As to total amount of serum to be administered.*—Continued injections at short intervals are required until definite improvement and inhibition of growth of membrane results. In severe naso-pharyngeal and laryngeal cases, 40,000 units may be found necessary. In many cases no improvement results until 20,000 units have been given.

*Method of Injection.*—Injection must in all cases be made with aseptic precautions. The skin must be carefully cleaned and prepared, and the needles and syringes kept aseptic. The serum may be injected in the flank or, better still, into the loose tissue between or below the shoulder blades where the skin is little sensitive and the patient cannot see what is going on. An antitoxin syringe should be always kept in readiness by medical practitioners, so that antitoxin may be administered without delay in doubtful cases.

